# Effects of fenobucarb based-Excel Basa 50EC on brain cholinesterase of juvenile snakehead fish (*Channa striata*) in the Vietnamese Mekong Delta’s rice fields

**DOI:** 10.5620/eaht.2023027

**Published:** 2023-12-28

**Authors:** Huynh Van Thao, Bui Thi Chuyen, Pham Van Toan, Tran Sy Nam, Nguyen Van Cong

**Affiliations:** 1 College of Environment and Natural Resources, Can Tho University, Vietnam; 2 United Graduate School of Agricultural Science, Tokyo University of Agriculture and Technology, Japan

**Keywords:** *Channa striata*, cholinesterase, fenobucarb, rice field exposure, toxicology

## Abstract

Fenobucarb is one of most common insecticides applied to rice crops in the Vietnamese Mekong Delta. Paddy fields are preferred habitats for snakehead fish (*Channa striata*). Therefore, the probability of exposure risks and growth effects is highly. This paper aimed to examine the effects of using fenobucarb based – Excel Basa 50EC on the brain cholinesterase (ChE) of snakehead fish. Two rice fields, in which a single dose of Excel Basa 50EC was applied to one field, whilst the other acted as a control. Each field was subdivided into three plots by earthen dikes. In each plot, one fish cage (1.2 m x 1.2 m x 1.2 m) was installed that was stocked with 20 snakehead fish. The Excel Basa 50EC was applied once at the indication dose. The results highlighted that the concentration of fenobucarb in water at 1 hr after application was 116.72 ppb ± 12.64, which decreased to 23.96 ± 6.61 ppb after d and then to below detection limits (0.02 ppb). For fish living in this field, no mortality was seen, but ChE was significantly inhibited for 31 % on the first day and recovery following 7 days of exposure. Residues of fenobucarb in soil and fish should also be investigated furthermore.

## Introduction

Vietnamese Mekong Delta (MD) is the rice bowl of Vietnam. Although the total area of the VMD accounts for approximately 12 % of the total national area, this region yearly produces more than 50 % of the rice production of the whole of Vietnam. In VMD, rice is planted from two to three crops per year. Therefore, a large amount of land area here is disturbed for rice cultivation. It is noticeable that pesticides have been repeatedly applied to rice paddies to prevent crop damage by insects. Farmers in the VMD have commonly applied pesticides 5 to 8 times per rice crop (approximately 100-day growth duration), of which approximately 50 % of farmers have frequently used pesticides with higher than permissible levels of producers [1]. Fenobucarb is a widely utilized active ingredient, which belongs to the carbamate group. The active is classified within toxic group II. In 2020, there were 39 commercial pesticides containing fenobucarb on the Vietnamese market for purchase [2]. Excel Basa 50EC, containing 50 % fenobucarb by weight, is the prevailing usage in the VMD.

Fenobucarb has been found to inhibit the enzyme cholinesterase (ChE) involved in regulating nerve impulse transmission in animals. In most aquatic species, a ChE inhibition of 70 % results in death [3], with an inhibition rate of 30 % considered the maximum allowable threshold without affecting the organism's health [4]. Tam et al. [5] found that the concentration of fenobucarb after application in the VMD’s rice fields was 81.7 ± 28.3 ppb, causing significant ChE inhibition in fish climbing perch (*Anabas testudineus*). In the VMD, the concentration of fenobucarb in surface water, and soil and sediment varies approximately 0.11 – 5 μg l^–1^ and 1.7 – 4.3 μg kg^–1^ [6].

Snakehead (*Channa striata*) is a vital fish species for protein sources of the people in the VMD. The snakehead prefers to live in shallow ponds and swamps that dominate aquatic plants and muddy surfaces. Snakehead is a carnivorous fish with dagger-like teeth that can eat zooplankton, larvae, beetles, fish, insects, frogs, and crustaceans. However, the species is under pressure owing to overexploitation and habitat loss [7]. Although the investigation on the wild population sizes or productivity in the rice fields remained unclear, its population size is presently reduced by approximately 30 – 60 % compared to the last 4 decades [8]. The fish distributes in various water bodies, including rivers, canals, ponds, and rice fields [9]. Therefore, the fish is at high risk of exposure to the use of pesticides for rice in this region. Under laboratory conditions, 96h-LC50 of fenobucarb for juvenile snakehead fish was 11.4 mg L-1 and brain ChE was significantly inhibited at sublethal concentrations (1 % LC50-96h) [10]. In the rainy season, using pesticides containing fenobucarb caused significant effects on the ChE of snakehead fish [11]. Farmers in the VMD typically cultivated rice from two to three crops per year. Environmental conditions such as temperature, rain regime, and sunlight intensity vary over seasons and may result in different effects on organisms. In this study, the effects of using Excel Basa 50EC containing 50 % weight-based fenobucarb for rice in the dry season on snakehead fish were investigated. Findings from the study contribute to the literature on the risks of fenobucarb on snakehead fish as well as for other aquatic species.

## Materials

### Chemicals

Insecticide Excel Basa 50EC, which contains 50 % active ingredient fenobucarb produced by the Saigon Plant Protection Joint Stock Company, was used for exposure.

Sodium hydrogen phosphate dihydrate (NaH_2_PO_4_.2H_2_O) and di-sodium hydrogen phosphate dihydrate (Na_2_HPO_4_.2H_2_O), purchased from Merck, as used to prepare buffer solution with pH 7.4 and pH 8.

Chemical 5,5’-dithiobis (2 nitrobenzoic acid) (C_14_H_8_N_2_O_8_S_2_), also termed DTNB, was produced by Sigma Aldrich, Germany. Acetylthiocholine iodide, acetone and distilled water were used to wash the sample grinding machine.

### Organisms

Snakehead fishes (4 – 5 g individual-1, 15 – 18 cm, 75 days old) were purchased from a hatchery in Can Tho City. Fish were placed into a 600-L composite tank with tap water for 2 weeks to adapt to laboratory conditions (pH 6.7 ± 0.05, water temperature 26.5 °C ± 0.4). Water was continuously aerated to maintain dissolved oxygen above 4 mg L–1. Tank water was changed (10% volume) once per day. Fish were fed twice a day with industrial pellets (1.5 mm tablet–1). Healthy fish (active, no signs of disease or infection) were selected for the experiment.

## Methods

### Experimental design

Two large rice fields with approximately 9,000 m^2^ surface area per field in Binh Thuy district, Can Tho province, were utilized in this study. One field was used as a control treatment applied without pesticide, whilst the insecticide fenobucarb was applied to the remaining field. Both fields were sown with IR504 rice variety at a density of 250 kg ha^–1^. The water level within the fields was kept in the range of 10 - 15 cm for the duration of the experiment. Within each field, earthen dikes were established to separate the field into three equal plots (approximately 3,000 m^2^ plot^–1^). Three replications were performed to avoid water leakage between plots during the experiment. Within each plot, rice plants were removed to install a fish cage (1.2 m x 1.2 m x 1.2 m in response to the wide x length x high). The cage was made from nylon mesh. These cages were left for 4-day sedimentation. Thereafter, twenty healthy snakehead fish, similar size were carefully removed from the tanks and placed into each cage. Fish were fed daily at 09:00 AM by commercial pellets. After seven days of stocking fish into the cages to adapt to the water environment, farmers were provided with the target pesticide (Excel Basa 50EC) to spray according to the instructions on the label of the producer (2.0 L^–1^ ha^–1^ for Excel Basa 50EC). The pesticide was applied to rice at the 35-day stage after sowing. The pesticides were only applied once during the whole experiment.

Water samples were collected on the fields (*i*) at the time just before pesticide application, (*ii*) 1 h and (*iii*) 1, 3, 5, 7, and 14 days after spraying using one-litter dark glass bottles with PTFE sealed caps with three replications in each field (control and fenobucarb-sprayed fields). These samples were stored in a foam box added with ice. Collected samples were detected with the actual concentration of fenobucarb using GC-MS [12]. Fenobucarb residues were quantified using a gas chromatograph (GC) (Shimadzu GC - 2010, Japan) coupled with a mass selective detector (MS) (Shimadzu MS - QP2010) and equipped with a Shimadzu AOC – 20S automatic sampler. The GC was fitted with a Rxi@5Sil MS W/Inter fused silica capillary column: 30 m length × 0.25 mm ID × 0.5 μm film thickness. Helium was used as carrier gas with a constant flow rate of 1.0 mL min−1. The following oven temperature program was applied: i) 70 °C initial temperature for 1.0 min; ii) increase at 15 °C min−1 to 200 °C and keep for 5 min; iii) increase at 8 °C min−1 to 300 °C, keep for 10 min. The injector block temperature was set up to 250 °C. The injection volume was 1 μL. The water properties of water level, temperature, pH, and DO were checked every two days from 6:30 to 7:30 and 14:00 to 15:00 at three points within each cage.

Fish in cages were collected (*i*) 1 day before spraying and (*ii*) 1, 3, 5, 7, and 14 days after pesticide application for ChE assay. Two fish were collected from each cage (six fish/field). Fish-collected samples were sealed plastic bags placed into an icebox to be killed quickly. It was then transported to the laboratory for processing ChE analysis.

### Sample preparation

Fish was first individually checked for its weight and length. Then, the brain of each fish was separated by cutting along the mouth to the fish's gills, then cutting across the middle of the skull to separate the skull. The brain was gently removed and placed in a 1.5 mL pre-weighed plastic Eppendorf. The Eppendorf containing the brain was then weighed again to determine brain mass. Each brain was ground in 0.1M phosphate buffer pH 7.4. After the sample solution was well mixed, 1 mL of the solution was removed and added to Eppendorf and then centrifuged at 2,000 rpm for 20 min at 4 °C. The upper phase of the solution after centrifugation was used for the ChE assay. After grinding, the grinder was washed with distilled water and acetone according to the following procedure: distilled water - acetone - distilled water.

### ChE assay

ChE activity was determined by using a spectrophotometer (Hitachi UH-5300) at 412 nm for 200 s based on the method of Ellman et al. [13], modified by Cong et al. [14]. Analytical steps were as follows: add 2.65 mL of 0.1M Phosphate pH 7.4 into the cuvette, then add 0.1 mL of DTNB solution and 0.05 mL of acetylthiocholine iodide solution; thereafter, 0.2mL of the centrifuged brain sample was added and measuring process was started. The blank was prepared similarly to the sample, but 0.2 mL of the brain sample was replaced by 0.2 mL of 0.1 M phosphate buffer pH 7.4. The measuring result was recorded when the correlation coefficient (r2) reached 0.9 or more.

ChE activity of ChE enzyme is calculated as the following equation:


(1) $$\mathrm{HT}=\frac{A\times C_{V}\times H_{V}}{F\times L\times S_{V}\times P_{V}}$$

Where, A: sample adsorption in 1 min (Abs/min); Cv: cuvette volume (mL); Hv: volume of buffer solution used to grind the sample (mL); F: coefficient (13.6); L: Cuvette length (cm); Sv: sample volume after centrifugation (mL); Pv: mass of ground sample (g).

Enzyme inhibition ratio (%):


(2) $$\mathrm{IR}=100-100\frac{A}{\mathrm{Adc}}$$

Where, IR: inhibition rate; A: ChE activity measured in each sample (µ M g^–1^ min^–1^); Adc: average ChE activity in the control treatment (µ M g^–1^ min^–1^).

### Data processing

Data were statistically analyzed using the RStudio software package (The R Foundation for Statistical Computing, R version 4.1.3 for Windows). The ChE activity data were tested for normal distribution and homogeneity of variance before statistical analysis. Analysis of variance and the Duncan test were applied to compare treatments with controls and among treatments with a statistically significant difference at 95 % (p<0.05).

## Results and Discussion

### Variations of water temperature, pH, and dissolved oxygen

Water temperature in the control paddy field plots ranged from 28.0 ± 0.03 to 30.6 ± 0.12 °C, while in the pesticidesprayed field, it varied between 27.7 ± 0.07 and 30.6 ± 0.09 °C (Table 1). In the morning, the temperature was lower than in the afternoon measurements. The temperature difference between the treatments was less than 1° C. The sunlight intensity causes the difference in temperature between morning and afternoon. An average temperature range of 27.7 to 30.6 °C is suitable for the survival of snakehead fish [15]. According to Cong et al. [16], the temperature from 23 - 36 °C did not affect the ChE activity of snakehead fish. In this experiment, the temperature difference between morning and afternoon was not more than 3 °C.

The water pH value in the experiment plots was quite stable, ranging from 6.6 ± 0.05 to 6.6 ± 0.07 in the morning and from 6.7 ± 0.01 in the afternoon. This shows that the pH was uniform among the experimental plots. According to Lee and Ng (1994), snakehead fish can tolerate a wide pH range, from 4.25 to 9.4. Therefore, the pH value in the experimental fields was suitable for snakehead fish.

Dissolved oxygen in the experimental fields was low, ranging from 1.3 mg L^–1^ ± 0.05 to 2.0 ± 0.06 (mg L^–1^). The experiment was conducted when the rice was 45 days after sowing. At this time, the coverage area of rice leaves was high, so the diffusion ability of oxygen from the air into the water was limited. However, snakehead fish is an obligate airbreathing species [17]. It can survive in low DO conditions. According to Cong et al. [10], the ChE of snakehead fish is not affected by DO sufficiency (> 5 mg L^–1^) or DO deficiency (< 2 mg L^–1^).

### Fenobucarb water concentrations

Before spraying, fenobucarb concentration in water was all below the detection limit (0.02 ppb) in both the control and the later pesticide-sprayed fields (Table 2). One hour after spraying, the concentrations of fenobucarb increased by 116.72 ± 12.64 ppb in the pesticide-sprayed field. After 1 d of spraying, the concentration of fenobucarb rapidly decreased to 23.96 ± 6.61 ppb; thereafter, no further detection was found. Tam et al. [5] found that fenobucarb was detected only one hour after applying fenobucarb (Bassa 50EC) for rice with a water concentration of 81.7 ± 28.3 ppb, lower than residuals in this study. Fenobucarb has a water solubility of 420 mg L^–1^ (20 °C), coefficient K_ow_ of 2.78, and coefficient K_oc_ of 1.068 (http://sitem.herts.ac.uk). Due to these physio-chemical properties, fenobucarb could adhere to suspended particles and settle after spraying. This may be one reason for the rapid decrease in the fenobucarb concentration in water. The rapid loss of fenobucarb may also have been due to the presence of microorganisms capable of degrading the pesticide ingredient. For example, it was reported that in water taken from rice fields with a history of diazinon treatment, diazinon concentrations fell much more rapidly than in rice fields which had never received diazinon [18]. Fenobucarb was used popularly for rice in the VMD [1, 19]. Therefore, within rice fields in the VMD, it is expected that microorganisms exist capable of degrading fenobucarb. However, this hypothesis requires further investigation.

### Effects of using fenobucarb based – Excel Basa 50EC on the activity of brain ChE in snakehead fish

**Figure 1.**
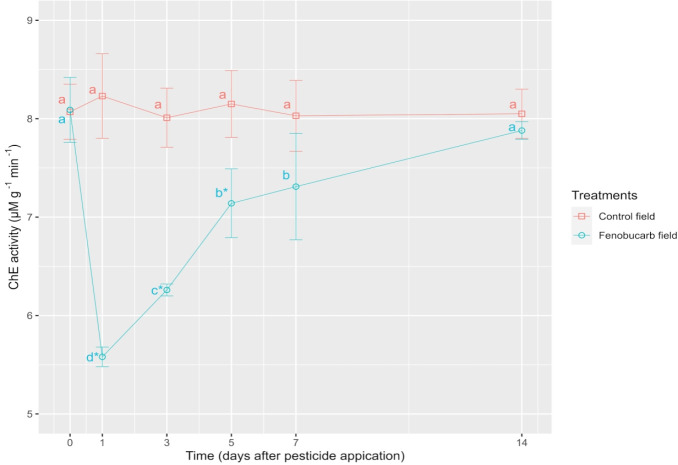
Variation in the brain ChE activity of snakehead fish in the control rice field plots and in the fenobucarb sprayed rice field plots. Data present mean ± SD, n=6. At the same time, an asterisk (*) indicates a significant difference in control activity. In the same line, the same letter indicates an insignificant difference among (p>0.05)

Fish size was an insignificant difference between the controlled and treated fields. Mean fish weight was 4.4 ± 0.6 g in the controlled treatment and 4.3 ± 0.7 g in the pesticide treatment. For length, it was 15.5 ± 2.4 cm in the controlled field and 15.4 ± 2.8 cm in the treated field. Fish mortality was not seen in both the control and the fenobucarb sprayed plots during the experimental period. In the field sprayed with fenobucarb, the activity of ChE decreased greatly to 5.59 ± 0.10 μM g-1 min-1 (i.e., 31 % inhibition rate) after 1 day of spraying, thereafter gradually recovering (Figure 1). The activity of ChE was not different from the control from the 14th day onwards after spraying.

Fenobucarb belongs to the carbamate pesticide group, and its mode of action is ChE inhibition [20]. Fenobucarb is moderately toxic to snakeheads with LC50-96h 11.6 ppm [16]. The water concentration of fenobucarb in the experimental plots was far lower than LC50-96h. Therefore, no mortality appeared during the experiment.

Under laboratory conditions, fenobucarb was found to cause quick ChE inhibition, followed by rapid recovery for snakehead fish *C. striata* [16]. In this present study, the brain ChE of snakehead fish greatly declined on day 1 after pesticide application and gradually recovered but remained lower than that of the control field plots up to the 7th day after spaying. Snakehead fish is an obligate air-breathing species [17]; Dissolved oxygen in the present study was very low, varying from 1.3 ± 0.05 mg L^–1^ to 2.0 ± 0.06 mg L^–1^ (Table 1). In low DO conditions, snakehead fish may shift to air-breath. This action would reduce fenobucarb intake and cause low ChE inhibition. Cong et al. [21] found that snakehead fish avoid pesticides by increasing air breathing in low concentrations of diazinon (1 % LC50-96h).

Tam et al. [5] found that ChE activity of air-breathing climbing perch (*Anabas testudineus*) after one-day spraying fenobucarb at the indicated dose inhibited 26 % of control activity, with full recovery seen at day 3 post spraying. Following exposure to organophosphate diazinon, snakehead fish *C. striata*'s ChE significantly declined to 21 d with no sign of full recovery [21]. The LC50-96 h of fenobucarb for snakehead fish is 14 times higher than that of diazinon [10]. It is indicated that fenobucarb is less toxic to snakehead fish than diazinon.

Fulton and Key [3] found that most aquatic organisms died when their ChE was inhibited by more than 70%, and abnormal activities were found when their ChE was inhibited between 30 and 70 % of normal values. Apreal et al. [4] suggested the limitation for ChE inhibition is 30 %. In the present study, the maximum ChE inhibition was 31 %, and no fish mortality was observed. However, the significant inhibition of ChE up to 7 d after using Excel Basa 50EC was observed. The pesticide was applied 5 – 7 times per cop (100 days). Therefore, the fish species may just fully recover and then continue to be exposed to the next pesticide application. Water concentration of fenobucarb was less than the detection limit (0,02 ppb) after 1 day post spray. Although fenobucarb was less than the detection limit in water, it may remain present in the sediments, causing exposure to fish. This assumption should be investigated for a clear explanation. It means that biological parameters such as ChE can be used to indicate the exposure of fish to pesticide application. The findings from the present study indicate that the use of fenobucarb for rice did not result in any serious effects on snakehead fish such as no mortality, low ChE inhibition, and quick recovery. However, in the rice paddy field ecosystem, many other aquatic species such as phytoplankton, zooplankton and zoobenthos coexist [22]. These are important natural foods for snakehead fish, particularly at larval stages. Snakehead fish reproduce many times a year, but the main season is in the wet season [23]. In the wet season, approximately 50 % of the reproduction of snakehead fish was found in the paddy field [24]. Young early-stage snakehead fish may also be more sensitive to toxicity than older fish. Therefore, the effects of using rice fields on the early stage of snakehead fish, as well as plankton and benthos, should be considered in further studies.

## Conclusions

Applying Excel Basa 50EC for rice at the instructed dose was found to not cause mortality for snakehead fish *C. striata* at fingerling size. However, the brain ChE of the exposed snakehead fish was significantly lower than the normal value up to 7 d post application, although fenobucarb in water was below the detection limit (0.02 ppb) after one day of spraying. Our findings suggest further studies on fenobucarb residues in soil and fish are required to better understand the distribution of pesticide residues after spraying and assess the safety of snakehead fish for human consumption. Moreover, the effects of using fenobucarb for rice cultivation on the early stage of snakehead fish, plankton and benthos should also be further considered.

## Figures and Tables

**Table 1.** Variation of environmental parameters during the experimentation.

Parameters	Control field		Pesticide field	
	*Morning*	*Afternoon*	*Morning*	*Afternoon*
Temperature (°C)	28.0 ± 0.03	30.6 ± 0.12	27.7 ± 0.07	30.6 ± 0.09
pH	6.6 ± 0.05	6.7 ± 0.01	6.6 ± 0.07	6.7 ± 0.01
DO (mg L^–1^)	1.3 ± 0.06	1.8 ± 0.04	1.3 ± 0.05	2.0 ± 0.06
Water level (cm)	14.5 ± 0.02	13.7 ± 0.02	14.2 ± 0.05	13.3 ± 0.16

**Table 2.** Fenobucarb concentrations in water of the experimental rice fields.

Time	Fenobucarb (ppb)	
	*Control field*	*Pesticide field*
Before spraying	< DL	< DL
1 hour after spraying	< DL	116.72 ± 12.64
1 day after spraying	< DL	23.96 ± 6.61
3 days after spraying	< DL	< DL
5 days after spraying	< DL	< DL
7 days after spraying	< DL	< DL
14 days after spraying	< DL	< DL

Note: DL = Detection limit = 0.02 ppb. Data presented mean ± SD, n=3
